# Magnon Orbital Nernst Effect in Honeycomb Antiferromagnets without Spin–Orbit Coupling

**DOI:** 10.1021/acs.nanolett.4c00430

**Published:** 2024-04-29

**Authors:** Gyungchoon Go, Daehyeon An, Hyun-Woo Lee, Se Kwon Kim

**Affiliations:** † Department of Physics, Korea Advanced Institute of Science and Technology, Daejeon 34141, Korea; ‡ Department of Physics, Pohang University of Science and Technology, Pohang 37673, Korea

**Keywords:** magnon orbital moment, Nernst effect, thermal transport, honeycomb antiferromagnet, magnetoelectric effect

## Abstract

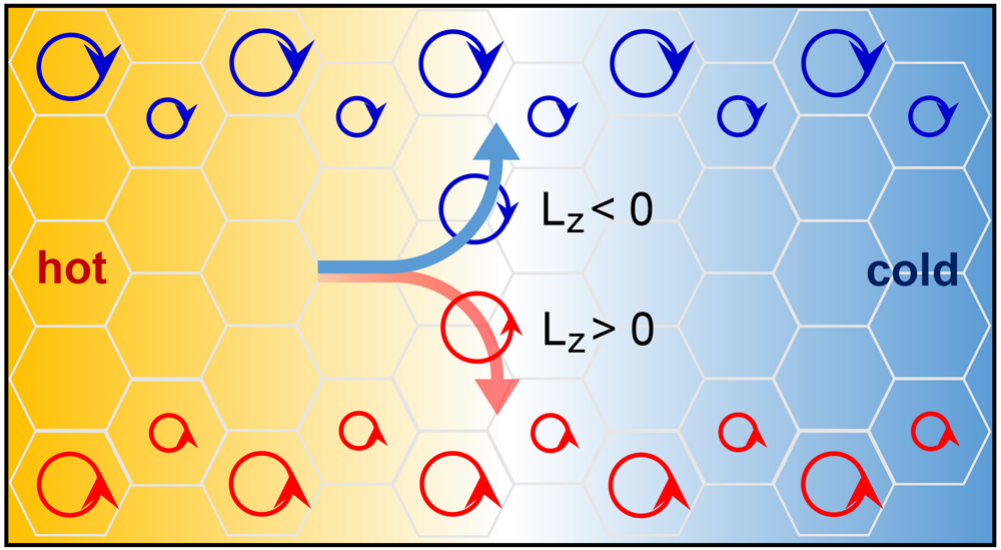

Recently, topological responses of magnons have emerged as a central theme in magnetism and spintronics. However, resulting Hall responses are typically weak and infrequent, since, according to present understanding, they arise from effective spin–orbit couplings, which are weaker compared to the exchange energy. Here, by investigating transport properties of magnon orbital moments, we predict that the magnon orbital Nernst effect is an intrinsic characteristic of the honeycomb antiferromagnet and therefore, it manifests even in the absence of spin–orbit coupling. For the electric detection, we propose an experimental scheme based on the magnetoelectric effect. Our results break the conventional wisdom that the Hall transport of magnons requires spin–orbit coupling by predicting the magnon orbital Nernst effect in a system without it, which leads us to envision that our work initiates the intensive search for various magnon Hall effects in generic magnetic systems with no reliance on spin–orbit coupling.

The collective low-energy excitations of the ordered materials are of great interest in condensed matter physics. One of the representative examples is a quantum of spin waves, called a magnon, which is a charge-neutral boson in magnetic materials. Magnons have been intensively studied for technological applications since they can realize Joule-heating-free information transport and processing.^1^ In addition, for fundamental interest, various topological properties of magnon bands have been investigated in the context of the magnon Hall effect^2−7^ and the spin Nernst effect.^8,9^ According to the existing theories, the finite Hall response of magnons can occur as manifiestations of spin–orbit coupling through the Dzyaloshinskii–Moriya interaction (DMI)^5−10^ or through the magnon–phonon coupling.^11−14^ The Hall response can also occur in spin texture systems with the scalar spin chirality^2,3^ or the long-range dipolar couplings,^15,16^ which act as effective spin–orbit couplings.

In electronics systems, on the other hand, there have been studies showing that electrons can exhibit a Hall effect *without* spin–orbit coupling, owing to their orbital degree of freedom.^17−20^ This discovery evoked a surge of interest in electron–orbital transport phenomena such as the orbital Hall effect^21−28^ and the orbital torque.^29−31^ Moreover, there have been theoretical suggestions that orbital-dependent electron transport critically affects electron spin dynamics when spin–orbit coupling is present. For instance, it has been suggested^19−21^ that the orbital Hall effect may play a crucial role in the spin Hall effect.

Motivated by the aforementioned advancement of our understanding of electron orbitals, the orbital motion of magnons has started garnering attention recently in magnetism and spintronics. For example, the circulating magnonic modes have been investigated in confined geometries such as whispering gallery mode cavities,^32−34^ magnetic nanocylinders and nanotubes.^35−38^ Also, the orbital magnetization of magnons has been pointed out as the origin of weak ferromagnetism in a noncollinear kagome antiferromagnet (AFM) with the DMI.^39^ Recently, the orbital-angular-momentum textures of the magnon bands have been realized in collinear magnets with nontrivial networks of exchange interaction.^40−42^ Furthermore, inspired by achievements in topological metamaterials,^43−45^ topological magnonic modes carrying the magnon current circulation have been demonstrated in honeycomb magnets with exchange-interaction modulation.^46^ However, despite the strong interest in magnon orbitals, studies on their transport properties are very limited.^39,40^ For example, the magnon orbital magnetic moment discussed in ref (39) is brought about by spin–orbit coupling (DMI) and therefore its physical manifestations are bound to be weak. Also, ref (40) focuses on the equilibrium magnon orbital angular momentum, with little discussion of its transport. Therefore, it is an open question whether the magnon orbital degree of freedom can induce a Hall phenomenon *without* spin–orbit coupling. If the answer is yes, then the Hall transport of the magnon can achieve significant magnitudes, as it is not constrained by the spin–orbit coupling. This would result in a substantial advancement in magnon-based spintronic devices.

In this work, we answer the question by investigating the transport of magnon orbital moments, which we define as **L̂** = 1/4(**r** × **v** – **v** × **r**) analogously to the modern theory of electron orbital magnetization.^47−49^ This magnon orbital moment represents a self-rotation of the magnon wave packet similar to the electron orbital magnetic moment,^24,25^ but differs from the electron counterpart in that it does not accompany the magnetization due to the vanishing electric charge of magnons. For the model system of 2D honeycomb AFMs, we demonstrate that the magnon orbital moments can exhibit a Hall effect, namely the magnon orbital Nernst effect, *without* spin–orbit coupling. Owing to strenuous efforts to realize magnetism in various 2D magnetic crystals, our proposal can be tested in a number of transition metal compounds known to host honeycomb AFMs, e.g., MnPS_3_, MnPSe_3_, and VPS_3_.^50−53^ The magnon orbital moment represents a magnon current circulation as shown in Figure 1a. We find that application of a longitudinal temperature gradient drives thermal magnons to opposite transverse directions depending on their orbital characters (see Figure 1b), giving rise to a magnon Hall phenomenon, the magnon orbital Nernst effect. To propose an experimental method for detecting the accumulation of the magnon orbital moment at the sample edges induced by the magnon orbital Nernst effect, we invoke the magnetoelectric effect by which a magnonic spin current induces an electric dipole moment. Since the magnon orbital moment can be regarded as a magnonic spin-current circulation, the aforementioned magnetoelectric effect dictates that the magnon orbital moment should engender a polarization charge in a two-dimensional space^4,54,55^ (see Figure 1c) and thus the magnon orbital accumulation should be accompanied by the accumulation of the polarization charge. We estimate the electrostatic potential profile induced by the magnon orbital moment accumulation.

**Figure 1:**
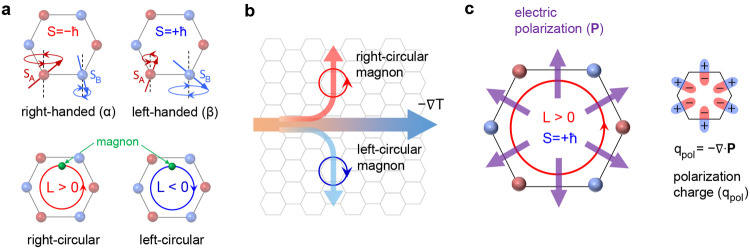
Magnon spin, orbital moment, and magnon-orbital-induced polarization. (a) Schematics of the magnon spin (*S*) and magnon orbital moment (*L*) in the honeycomb AFM. The magnon spin and the magnon orbital moment are determined by, respectively, the precessions of constituent spins within the sites and the intersite hopping. (b) Schematics of the magnon orbital Nernst effect, where a temperature gradient ∇*T* induces a net magnon-orbital-moment current in a transverse direction consisting of oppositely moving right-circular magnons and left-circular magnons. (c) Schematics of the polarization **P** induced by the circulating magnonic spin current in the case of orbital moment *L* > 0 and spin *S* = +*ℏ* (left) and the corresponding polarization charge *q*_pol_ = −∇·**P** (right). The sign of the polarization charge is determined by the product of the sign of the magnon spin *S* and the direction of the spin current circulation, i.e., the sign of *L*.

## Model Construction

**Figure 2:**
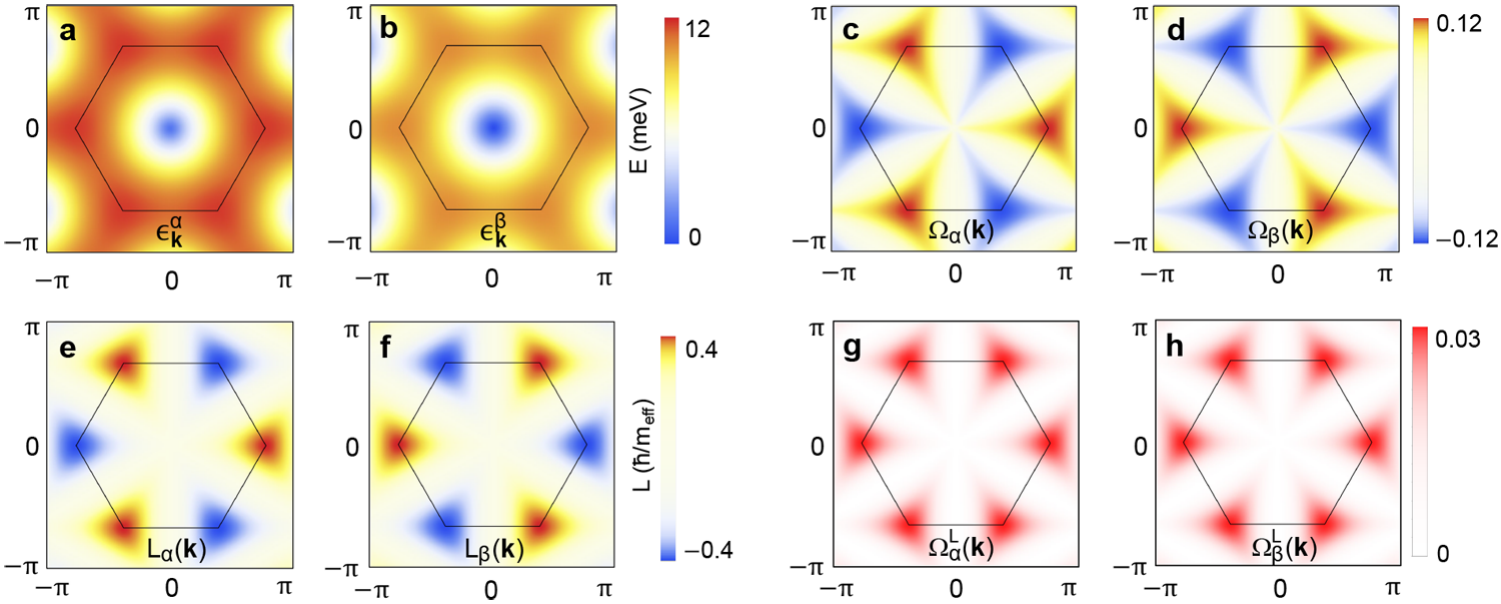
Characteristics of magnonic bands. (a, b) Magnon band structures [eq 4], (c, d) Berry curvatures [eq 5]. (e, f) Equilibrium orbital moment structures [eq 6]. (g, h) Orbital Berry curvatures [eq 7] of two magnonic states denoted by α and β. For material parameters, we take *J* = 1.54 meV and *KS* = 0.0086 meV, and *g*μ_*B*_*B* = 0.625 meV. For (e and f), *m*_eff_ ≈ – 71 *m*_*e*_ is the magnon effective mass at the *K*(*K*′)-points (*m*_*e*_ is the free electron mass).

Here we consider a 2D AFM on a honeycomb lattice
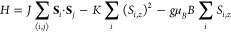
1where *J* (> 0) is the antiferromagnetic exchange coupling and *K* (> 0) is the eas*y*-axis anisotropy, *g* is the g-factor, μ_*B*_ is the Bohr magneton, and *B* is the applied magnetic field. In this study, we focus on the case where a ground state is the collinear Néel state along the *z*-axis. We emphasize that the easy-axis anisotropy and the external magnetic field are not essential for our main proposal if the perpendicular spin configuration is established. Note that our model does not include the (effective) spin–orbit couplings such as DMI and long-range dipolar couplings, and thus does not exhibit the magnon spin Nernst effect.^6−9^ Performing the Holstein–Primakoff transformation and taking the Fourier transformation, we have
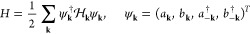
2with the momentum-space Hamiltonian
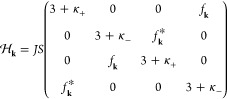
3where κ_±_ = (2*K* ± *g*μ_*B*_*B*/*S*)/*J* and  with , **a**_2_ = *a*(0, 1), and . The magnon excitations can be described by the generalized Bogoliubov–de Gennes equation in the particle-hole space representation.^12,56^ In this representation, the pseudoenergy-eigenvalue satisfies , where σ_3_ = diag(1, 1, −1, −1) is the Pauli matrix acting on the particle-hole space and ϵ(**k**) are the magnon bands given by
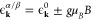
4where  and κ = 2*K*/*J* (see the Supporting Information). Here, the indices α and β stand for two magnonic bands with opposite spin angular momenta (see Figure 1a). The topological property of the magnonic state |*n*⟩ is characterized by the Berry curvature:^57,58^
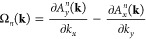
5where **A**^*n*^(**k**) = ⟨*n*|σ_3_*i∂*_**k**_|*n*⟩/⟨*n*|σ_3_|*n*⟩ is the Berry connection. Figure 2a–d shows the magnon band structures ϵ_**k**_^*n*^ and the corresponding Berry curvatures Ω_*n*_(**k**) with *n* = α, β. In the honeycomb AFM, the broken inversion symmetry allows a nonzero Berry curvature without the DMI. Also, because of the combined symmetry of the time-reversal  and a 180° spin rotation around the *x*-axis  of the Hamiltonian, the energy spectra are even in momentum space (ϵ_**k**_ = ϵ_–**k**_), whereas the magnon Berry curvatures are odd [Ω_*n*_(**k**) = −Ω_*n*_(−**k**)].^8^ Therefore, the momentum-space integration of the magnon Berry curvature weighed by the Bose–Einstein distribution is zero for each band, indicating the absence of the Hall transport.

## Magnon Orbital Nernst Effect

Although the momentum-space integration of the magnon Berry curvatures vanishes, the nonvanishing and **k**-odd structure of the Berry curvature opens up a possibility for topological transport of certain quantities. If there is a momentum-dependent quantity whose profile is also odd in **k**, then its Hall effect can be present. We show below that this is indeed the case for the magnon orbital moment since it holds the same symmetry property in the momentum space as the Berry curvature,^40^ i.e., **k**-odd in the presence of the  symmetry with broken inversion symmetry.

We define the magnon orbital moment operator as **L̂** = 1/4(**r** × **v** – **v** × **r**) analogously to the electron orbital moment in the modern theory for orbital magnetization.^47−49^ Note that this *orbital moment* differs from the *orbital angular momentum* by dimension of mass. Analogous to the well-known formula of the intrinsic orbital moment in electron systems, we read the expectation value of the magnon orbital moment^4,24,25,47−49,59^
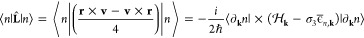
6which can be interpreted as the manifestation of the self-rotation of a wave packet within the wave packet formalism.^57^ The magnon orbital moment profiles  of the two magnons are shown in Figure 2e and f. The evaluated magnon orbital moment has the *C*_3_ rotation symmetry with **k**-odd structure In agreement with refs^40, 41^. Because *L*_*z*_^*n*^(**k**) = −*L*_*z*_^*n*^(−**k**) and ϵ_**k**_ = ϵ_–**k**_, the total magnon orbital moment is zero in equilibrium. However, our system can exhibit the intrinsic Nernst effect of the magnon orbital moment because both *L*_*z*_^*n*^(**k**) and Ω_*z*_^*n*^(**k**) are odd in **k**, and thus their product *L*_*z*_^*n*^(**k**) Ω_*z*_^*n*^(**k**) is **k**-even. Analogous to the generalized Berry curvature,^24,25,56,60^ we write the orbital Berry curvature which characterizes the magnon orbital Nernst transport as follows:
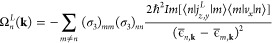
7where  is the magnon orbital moment current operator and  is the velocity operator. Note the summation is performed in the particle-hole space. The profiles of the orbital Berry curvatures of the two magnon modes are shown in Figure 2g,h. As expected, the profiles are even in **k** and thus their momentum-space integrations are finite, indicating the existence of the Hall effect of the magnon orbital moments with no (effective) spin–orbit coupling term such as the DMI. Also, the orbital Berry curvature remains finite when both *B* and *K* approach zero as long as the antiferromagnetic ground state is maintained. This reveals a unique characteristic of the orbital Berry curvature in honeycomb AFMs. In honeycomb AFMs, the magnon orbital moment and the Berry curvature of two magnonic bands are opposite to each other. Therefore, as *B* → 0, the net magnon orbital moment and Berry curvature become zero. However, even in such a case, the magnon orbital Berry curvature remains finite. Therefore, the magnon orbital Berry curvature and the resultant Nernst effect are inherent properties of the honeycomb AFM that originated solely from the exchange interaction and the lattice geometry.

**Figure 3:**
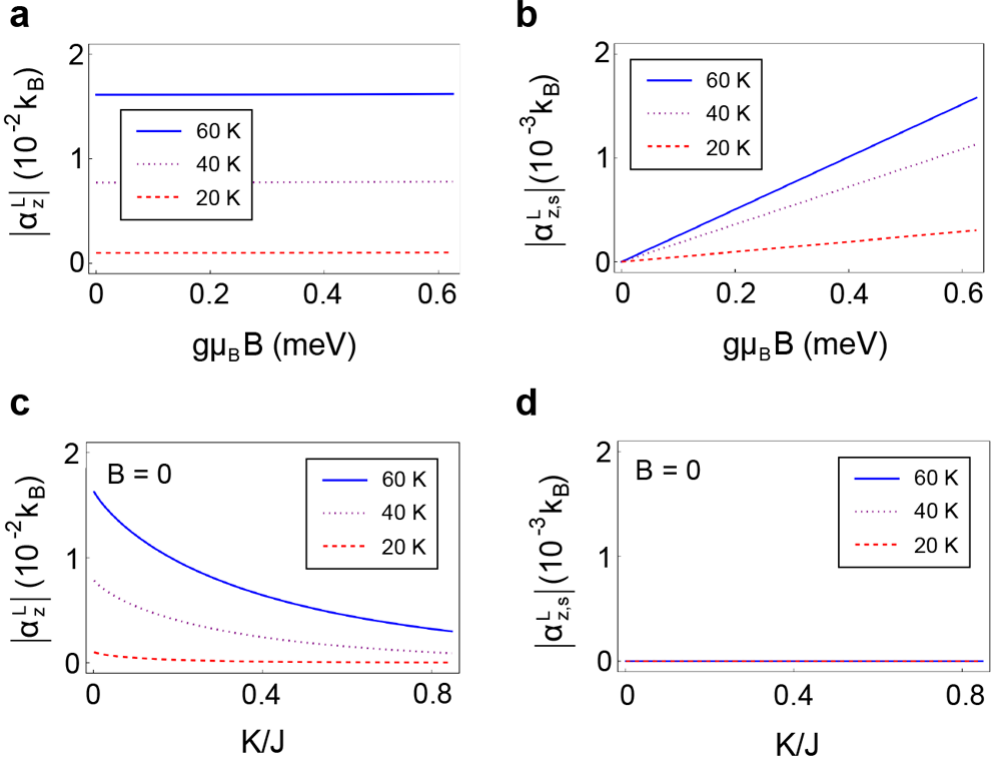
Magnon orbital Nernst conductivities. (a) Magnetic-field dependence of the magnon orbital Nernst conductivity α_*z*_^*L*^ and (b) the spin-polarized component of the magnon orbital Nernst conductivity α_*z*,*s*_^*L*^ = α_*z*,α_^*L*^ – α_*z*,β_^*L*^. (c) Anisotropy dependence of the magnon orbital Nernst conductivity and (d) the spin-polarized component of the magnon orbital Nernst conductivity. Here, *m*_eff_ ≈ −71 *m*_*e*_ is the magnon effective mass at the *K*(*K*′)-points (*m*_*e*_ is the free electron mass).

The magnon orbital Berry curvature leads to the transverse magnon orbital moment current in response to an external perturbation, namely the magnon orbital Nernst effect. The linear response equation of the transverse magnon orbital current driven by a temperature gradient is given by *J*_*m*_^OHE^ = −α_*z*_^*L*^∂_*x*_*T*,^9,56^ where α_*z*_^*L*^ = α_*z*,α_^*L*^ + α_*z*,β_^*L*^ is the magnon orbital Nernst conductivity with , where *k*_*B*_ is the Boltzmann constant, *c*_1_(ρ) = (1 + ρ) ln(1 + ρ) – ρ ln ρ, and ρ_*n*_ = (e^ϵ_*n*_/*k*_*B*_*T*^ – 1)^−1^ is the Bose–Einstein distribution. In Figure 3a, we show the orbital Nernst conductivity for different temperatures by using the material parameters of MnPS_3_: *J* = 1.54 meV and *KS* = 0.0086 meV.^61^ It is worth noting that a recent experimental report suggests that in the two-dimensional MnPS_3_, the DMI is negligible.^62^ Note that the orbital Nernst conductivity is almost independent of the magnetic field in Figure 3a, because the magnon eigenstates are unaffected by the magnetic field. Additionally, the orbital Nernst conductivity is finite even if both *B* and *K* approach zero (Figure 3c). This is our main result: The magnon orbital Nernst effect is an intrinsic characteristic of the honeycomb antiferromagnetic material and therefore, it manifests even in the absence of spin–orbit coupling.

## Magnon-Orbital-Moment-Induced Polarization

The magnon orbital Nernst effect induces the accumulation of the magnon orbital moment at the edges of a system. To propose an experimental method to detect the magnon orbital moment accumulation, here we develop a phenomenological model for the transverse electrostatic potential profile induced by the longitudinal temperature gradient via the magnon orbital Nernst effect. We emphasize that the following theory is qualitative in nature and thus intended to provide the order-of-magnitude estimation, not quantitative predictions. To begin with, let us review the relation between the spin current and the polarization. The spin current from a noncollinear spin configuration is known to induce an electric polarization by the combined action of the atomic spin–orbit interaction and the orbital hybridization:^54,55^
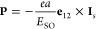
8where *e* is the magnitude of the electron charge, *a* is the distance between the two sites, **e**_12_ is the unit vector connecting two sites, **I**_*s*_ = *J*(**S**_1_ × **S**_2_) is the spin current from site 1 to site 2, and the energy scale *E*_SO_ is inversely proportional to the spin–orbit coupling strength. In the ground state of a collinear magnet, there is no spin current (**I**_*s*_ = 0) and thus no electric polarization (**P** = 0). However, the magnon consists of spatially varying noncollinear deviations from the ground state. Therefore, a magnon current in a collinear magnet gives rise to a finite spin current **I**_*s*_.^63,64^ By considering the typical energy scale of *E*_SO_, it has been predicted in refs (54, 55), and (65) that a measurable electric polarization can be induced by a magnonic spin current in magnetic materials. This magnetoelectric effect allows us to relate the magnon orbital motion, i.e., the circulating magnonic spin current, and the polarization charge density. We schematically depict the electric polarization produced by the magnonic spin-current circulation around a hexagon in a honeycomb lattice in Figure 1c. The magnonic spin-current circulation induces the electric polarization pointing outward or inward (and therefore the positive or negative polarization charge density), depending on the product of the magnon-spin sign and the magnon-orbital-moment sign.

Now let us consider the situation where the nonequilibrium accumulation of the magnon orbital moment is generated at the edges of the sample by a temperature gradient via the magnon orbital Nernst effect. In the absence of an external field along the *z*-direction, there would be a finite accumulation of the magnon orbital moment at the edges, but there would be no induced electric polarization, for spin-up magnons and spin-down magnons are equally populated and thus their contributions to the electric polarization cancel each other. However, when we apply an external magnetic field, spin-up magnons and spin-down magnons are populated unequally and thus the net spin density of magnons becomes finite. Consequently, the magnon orbital moment accumulation and the magnon orbital Nernst current are spin-polarized in the presence of the external field. In particular, the spin-polarized component of the magnon orbital Nernst conductivity α_*z*,*s*_^*L*^(= α_*z*,α_^*L*^ – α_*z*,β_^*L*^) is zero when the magnetic field is zero and becomes finite as the magnetic field is applied as shown in Figure 3b.

**Figure 4:**
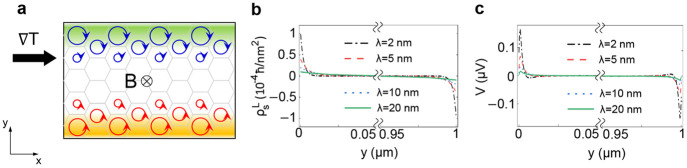
Magnon orbital moment accumulation and induced electric potential. (a) Schematic illustration of magnon orbital moment accumulation (red and blue arrows) and induced electric potential (background colors). (b) Calculation results of spin-polarized nonequilibrium magnon orbital moment accumulation ρ_*s*_^*L*^ and (c) the electric potential *V* induced by the nonequilibrium polarization.

Instead of the magnon orbital moment accumulation, what is directly related to the observable electric polarization is the spin-polarized magnon orbital moment accumulation ρ_*s*_^*L*^ = ρ_α_^*L*^ – ρ_β_^*L*^. To estimate the spin-polarized magnon orbital moment accumulation that is induced by the magnon orbital Nernst effect, we use the phenomenological drift-diffusion formalism by following the previous studies on electron orbital transport^23,28,66^ (see the Supporting Information). For parameters, we use *g*μ_*B*_*B* = 0.425 meV, *E*_SO_ = 1 eV, *T* = 30 K, and ∂_*x*_*T* = 1 K/μm with the constant magnon orbital relaxation time τ = 5 ns and various values of the magnon orbital diffusion length (see the Supporting Information for the potential profiles by using other values of τ). The considered system size is 1 μm × 1 μm. Figure 4b shows the resultant spin-polarized magnon orbital moment accumulation along the *y*-direction. We also numerically compute the electrostatic potential profile induced by the spin-polarized magnon orbital moment accumulation based on a simplified model for the inhomogeneous magnon orbital moment accumulation (see the Supporting Information), which is shown in Figure 4c. The accumulation of the magnon orbital moment gives rise to the electric potential profile via the magnetoelectric effect, which can serve as a probe of the proposed magnon orbital Nernst effect.

In this work, we have investigated the transport of magnon orbital moments in a honeycomb AFM and found that the finite magnon orbital Berry curvature gives rise to the magnon orbital Nernst effect. We emphasize that the magnon orbital Berry curvature does not require DMI or noncollinear spin textures with scalar spin chirality and, therefore, the magnon orbital Berry curvature is an inherent property of the honeycomb AFM originating solely from the exchange interaction and the lattice geometry. We also remark that, although we have considered a temperature gradient as a means to drive a magnon transport, one can also use electronic means to pump magnons by using the spin Hall effects.^67,68^

It is noteworthy that, here we consider the magnon orbital moment, not the magnon orbital angular momentum in refs (40−42). because of, first, the pragmatic benefit of the former coming from its close analogy to the modern theory for electronic orbital magnetization, and, second, the gauge-dependence of the latter and the resultant challenge to obtain a gauge-independent expression for the corresponding transport quantity. Additionally, we acknowledge that linear response expressions require the assumption that the corresponding operator commutes with the position operator.^9,56^ However, since the orbital moment incorporates a momentum operator [**L**, **r**] ≠ 0, additional current and source contributions arise due to this nonzero commutator, supplementing those outlined in refs (9) and (56). Nonetheless, formulating a comprehensive thermal transport theory that considers all contributions presents a challenge beyond the scope of our current study. Therefore, here we neglect the extra contributions stemming from the nonzero commutator [**L**, **r**]

For experimental schemes, we have discussed the magnon orbital moment accumulation detection through the electrical means by invoking the magnetoelectric effect. To detect electrostatic potential arising from magnon orbital moment accumulation, local probe measurement schemes such as scanning tunneling microscopy^69^ or Kelvin probe force microscopy^70^ can be used. Developing a scheme for electronic detection of magnon orbital-related phenomena in metallic magnets or conducting heterostructures is a potential avenue for future research. We acknowledge that the bulk spin current induced by the temperature gradient can also contribute to the bulk electric polarization. However, in this work, we consider the cases where the magnon spin current is blocked at the sample boundaries either by substrates or by open boundary conditions. In the considered situation, the bulk spin current caused by the thermal bias is effectively canceled out by the backflow spin current induced by the magnon accumulation gradient when the magnon diffusion length (*l*_*m*_) significantly exceeds the sample length (*L*). Consequently, the proposed experimental setup would work under the geometrical condition *l*_*m*_ ≫ *L*, and the temperature gradient control in the micrometer-scale 2D magnets can be achieved by utilizing a magnon valve structure with several electrodes.^71^ It is also noteworthy that, while the magnon-orbital Nernst effect does not necessitate spin–orbit coupling and an external magnetic field, the proposed detection scheme requires both spin–orbit coupling and an external magnetic field. Further investigation is necessary for a measurement setup operating without these requirements.

We here note that, while we focus on the electric polarization as the detectable manifestation of the magnon orbital moment in this work, there are several other degrees of freedom that are expected to couple with it such as photons with angular momentum and chiral phonons as mentioned in ref (40). We would like to emphasize that the magnon “orbital moment” is not directly related to the magnon “orbital angular momentum” unlike electrons and phonons for which the two quantities are naturally related. This point sets magnons apart from electrons and phonons, the significance of which is yet to be explored. It is worth mentioning that the magnon orbital moments are expected to be nonconserved unlike spin that is conserved in the absence of spin–orbit coupling. While the diffusion length of the magnon orbital moment is expected to be significantly shorter than the spin diffusion length, the detailed investigation of the diffusion of the magnon orbital moment is beyond the scope of the current work.

## Supporting Information Available

Calculation details of the model Hamiltonian, Berry curvature, magnon orbital Berry curvature, and induced electric polarization (PDF)
