# Supplementary material for: Magnon Orbital Nernst Effect in Honeycomb Antiferromagnets without Spin–Orbit Coupling
Source: Nano Lett. 2024 Apr 29;24(20):5968–74. doi: 10.1021/acs.nanolett.4c00430 (PMC11117403; doi:10.1021/acs.nanolett.4c00430)
Supplement: Supplementary file 1 — nl4c00430_si_001.pdf [file nl4c00430_si_001.pdf]

Supporting Information: Magnon Orbital Nernst Effect in Honeycomb Antiferromagnets without Spin-Orbit Coupling

Gyungchoon Go,[†] Daehyeon An,[†] Hyun-Woo Lee,[‡] and Se Kwon Kim[*,†]

†*Department of Physics, Korea Advanced Institute of Science and Technology, Daejeon 34141, Korea*

‡*Department of Physics, Pohang University of Science and Technology, Pohang 37673, Korea*

E-mail: sekwonkim@kaist.ac.kr

A model Hamiltonian of an antiferromagnet

In momentum space representation, our model Hamiltonian is

$$H = \frac{1}{2}\sum_{\mathbf{k}} \psi_{\mathbf{k}}^\dagger \mathcal{H}_{\mathbf{k}} \psi_{\mathbf{k}}, \qquad \psi_{\mathbf{k}} = (a_{\mathbf{k}}, b_{\mathbf{k}}, a_{-\mathbf{k}}^\dagger, b_{-\mathbf{k}}^\dagger)^T,$$

$$\mathcal{H}_{\mathbf{k}} = JS \begin{pmatrix} 3+\kappa_+ & 0 & 0 & f_{\mathbf{k}} \\ 0 & 3+\kappa_- & f_{\mathbf{k}}^* & 0 \\ 0 & f_{\mathbf{k}} & 3+\kappa_+ & 0 \\ f_{\mathbf{k}}^* & 0 & 0 & 3+\kappa_- \end{pmatrix}, \tag{S1}$$

where $\kappa_\pm = (2K \pm g\mu_B B/S)/J$ and $f_{\mathbf{k}} = \sum_j e^{i\mathbf{k}\cdot\mathbf{a}_j}$. As discussed in Ref.[1], the Hamiltonian is composed of two block diagonal matrices. Explicitly, we have

$$\mathcal{H}_{I,\mathbf{k}} = \begin{pmatrix} 3+\kappa_- & f_{\mathbf{k}}^* \\ f_{\mathbf{k}} & 3+\kappa_+ \end{pmatrix}, \tag{S2}$$

and $\mathcal{H}_{II,\mathbf{k}} = (\mathcal{H}_{I,\mathbf{k}})^*$. To diagonalize the Hamiltonian, we find the paraunitary matrix $U_{\mathbf{k}}$ satisfying $E_{\mathbf{k}} = U_{\mathbf{k}}^\dagger \mathcal{H}_{\mathbf{k}} U_{\mathbf{k}} = \mathrm{diag}(\epsilon_{\mathbf{k}}, \epsilon_{-\mathbf{k}})$. Following the notation used in Ref.[2], we write the eigenvalue equations

$$\begin{aligned} \sigma_3 \mathcal{H}_{\mathbf{k}} |u^R_{n,\mathbf{k}}\rangle &= \bar{\epsilon}_{n,\mathbf{k}} |u^R_{n,\mathbf{k}}\rangle, \\ \langle u^L_{n,\mathbf{k}}| \sigma_3 \mathcal{H}_{\mathbf{k}} &= \bar{\epsilon}_{n,\mathbf{k}} \langle u^L_{n,\mathbf{k}}|, \end{aligned} \tag{S3}$$

where $\sigma_3 = \mathrm{diag}(1,1,-1,-1)$ is the Pauli matrix acting on the particle-hole space. Here $\langle u^L_{n,\mathbf{k}}| = \langle u^R_{n,\mathbf{k}}|\sigma_3$ and $|u^R_{n,\mathbf{k}}\rangle = (U_{\mathbf{k}})_n$ are the left- and right-eigenvectors of the pseudo-Hermitian Hamiltonian $\sigma_3\mathcal{H}_{\mathbf{k}}$, respectively. The orthonormal relation of the eigenvectors reads $\langle u^L_{n,\mathbf{k}}|u^R_{m,\mathbf{k}}\rangle = \langle u^R_{n,\mathbf{k}}|\sigma_3|u^R_{m,\mathbf{k}}\rangle = (\sigma_3)_{nm}$ and the pseudo-eigenvalue satisfies $\bar{\epsilon}_{n,\mathbf{k}} = (\sigma_3\epsilon_{\mathbf{k}})_{nn}$. The energy eigenvalues of the magnon bands are

$$\epsilon^{\alpha/\beta}_{\mathbf{k}} = \epsilon^0_{\mathbf{k}} \pm g\mu_B B, \tag{S4}$$

where $\epsilon^0_{\mathbf{k}} = JS\sqrt{(3+\kappa)^2 - |f_{\mathbf{k}}|^2}$ and $\kappa = 2K/J$. Also, the paraunitary matrix $U_{\mathbf{k}}$ is given by

$$U_{I,\mathbf{k}} = \begin{pmatrix} \cosh(\theta_{\mathbf{k}}/2) & -\sinh(\theta_{\mathbf{k}}/2)e^{-i\phi_{\mathbf{k}}} \\ -\sinh(\theta_{\mathbf{k}}/2)e^{i\phi_{\mathbf{k}}} & \cosh(\theta_{\mathbf{k}}/2) \end{pmatrix}, \tag{S5}$$

and $U_{II,\mathbf{k}} = (U_{1,\mathbf{k}})^*$, where $\cosh(\theta_{\mathbf{k}}) = JS(3+\kappa)/\epsilon^0_{\mathbf{k}}$, $\sinh(\theta_{\mathbf{k}}) = JS|f_{\mathbf{k}}|/\epsilon^0_{\mathbf{k}}$, and $f_{\mathbf{k}} = |f_{\mathbf{k}}|e^{i\phi_{\mathbf{k}}}$. Note that the applied magnetic field only splits energy eigenvalues of the two magnonic states

and does not change $U_{\mathbf{k}}$.

Calculation details of Berry curvature and magnon orbital Berry curvature

We start from the Berry connection of the Bogoliubov–de Gennes Hamiltonian[3,4]

$$\mathbf{A}^n(\mathbf{k}) = \frac{i\langle u^L_{n,\mathbf{k}}|\partial_{\mathbf{k}}|u^R_{n,\mathbf{k}}\rangle}{\langle u^L_{n,\mathbf{k}}|u^R_{n,\mathbf{k}}\rangle} = (\sigma_3)_{nn}\langle u_{n,\mathbf{k}}|(i\sigma_3)\partial_{\mathbf{k}}|u_{n,\mathbf{k}}\rangle, \tag{S6}$$

here we use $\langle u^L_{n,\mathbf{k}}| = \langle u^R_{n,\mathbf{k}}|\sigma_3 \equiv \langle u_{n,\mathbf{k}}|\sigma_3$, and $\langle u_{n,\mathbf{k}}|\sigma_3|u_{n,\mathbf{k}}\rangle = (\sigma_3)_{nn}$. Then the Berry curvature is

$$\begin{aligned}\Omega_n(\mathbf{k}) &= \partial_{k_x} A^n_y(\mathbf{k}) - \partial_{k_y} A^n_x(\mathbf{k}) \\ &= i(\sigma_3)_{nn}\left[\partial_{k_x}\langle u_{n,\mathbf{k}}|(\sigma_3)\partial_{k_y}|u_{n,\mathbf{k}}\rangle - \partial_{k_y}\langle u_{n,\mathbf{k}}|(\sigma_3)\partial_{k_x}|u_{n,\mathbf{k}}\rangle\right] \\ &= i(\sigma_3)_{nn}\left[\left\langle \frac{\partial u_{n,\mathbf{k}}}{\partial k_x}\middle|\sigma_3\middle|\frac{\partial u_{n,\mathbf{k}}}{\partial k_y}\right\rangle - (k_x \leftrightarrow k_y)\right].\end{aligned} \tag{S7}$$

By inserting the identity operator $I = \sum_m (\sigma_3)_{mm}|u_{m,\mathbf{k}}\rangle\langle u_{m,\mathbf{k}}|\sigma_3$ into Eq. (S7) and using

$$\langle u_{n,\mathbf{k}}|\sigma_3\left|\frac{\partial u_{m,\mathbf{k}}}{\partial k_i}\right\rangle = \frac{\langle u_{n,\mathbf{k}}|\frac{\partial \mathcal{H}_{\mathbf{k}}}{\partial k_i}|u_{m,\mathbf{k}}\rangle}{\bar{\epsilon}_m - \bar{\epsilon}_n} \qquad (\text{for} \quad n \neq m) \tag{S8}$$

we have

$$\Omega_n(\mathbf{k}) = i\sum_{m\neq n}(\sigma_3)_{nn}(\sigma_3)_{mm}\frac{\langle u_{n,\mathbf{k}}|\frac{\partial \mathcal{H}_{\mathbf{k}}}{\partial k_x}|u_{m,\mathbf{k}}\rangle\langle u_{m,\mathbf{k}}|\frac{\partial \mathcal{H}_{\mathbf{k}}}{\partial k_y}|u_{n,\mathbf{k}}\rangle - (k_x \leftrightarrow k_y)}{(\bar{\epsilon}_m - \bar{\epsilon}_n)^2}. \tag{S9}$$

From Ref.[2], we read the expression of the magnon orbital Berry curvature

$$\Omega^L_n(\mathbf{k}) = 2\hbar^2\sum_{m\neq n}(\sigma_3)_{nn}(\sigma_3)_{mm}\frac{\mathrm{Im}\left[\langle u_{n,\mathbf{k}}|j^L_{z,y}|u_{m,\mathbf{k}}\rangle\langle u_{m,\mathbf{k}}|v_x|u_{n,\mathbf{k}}\rangle\right]}{(\bar{\epsilon}_{n,\mathbf{k}} - \bar{\epsilon}_{m,\mathbf{k}})^2}, \tag{S10}$$

where $v_i = \frac{1}{\hbar}\frac{\partial \mathcal{H}_{\mathbf{k}}}{\partial k_i}$ is the velocity operator and $j^L_{z,y} = \frac{1}{4}(v_y\sigma_3\hat{L}_z + \hat{L}_z\sigma_3 v_y)$ is the orbital current operator. By using the identity operator, we rewrite the interband matrix element in Eq. (S10) as

$$\langle u_{n,\mathbf{k}}|j^L_{z,y}|u_{m,\mathbf{k}}\rangle = \frac{1}{4}\sum_p (\sigma_3)_{pp}\left[\langle u_{n,\mathbf{k}}|v_y|u_{p,\mathbf{k}}\rangle\langle u_{p,\mathbf{k}}|\hat{L}_z|u_{m,\mathbf{k}}\rangle + \langle u_{n,\mathbf{k}}|\hat{L}_z|u_{p,\mathbf{k}}\rangle\langle u_{p,\mathbf{k}}|v_y|u_{m,\mathbf{k}}\rangle\right]. \tag{S11}$$

Then,

$$\Omega^L_n(\mathbf{k}) = \frac{\hbar^2}{2}\sum_{m\neq n}\sum_p (\sigma_3)_{nn}(\sigma_3)_{mm}(\sigma_3)_{pp}\mathrm{Im}\left[\frac{\langle u_{p,\mathbf{k}}|\hat{L}_z|u_{m,\mathbf{k}}\rangle\langle u_{n,\mathbf{k}}|v_y|u_{p,\mathbf{k}}\rangle\langle u_{m,\mathbf{k}}|v_x|u_{n,\mathbf{k}}\rangle}{(\bar{\epsilon}_{n,\mathbf{k}}-\bar{\epsilon}_{m,\mathbf{k}})^2} + \frac{\langle u_{n,\mathbf{k}}|\hat{L}_z|u_{p,\mathbf{k}}\rangle\langle u_{p,\mathbf{k}}|v_y|u_{m,\mathbf{k}}\rangle\langle u_{m,\mathbf{k}}|v_x|u_{n,\mathbf{k}}\rangle}{(\bar{\epsilon}_{n,\mathbf{k}}-\bar{\epsilon}_{m,\mathbf{k}})^2}\right]. \tag{S12}$$

By utilizing calculation procedure in Ref.[5–7], we obtain the matrix element of the magnon orbital moment

$$\begin{aligned}\langle u_{n,\mathbf{k}}|\hat{L}_z|u_{p,\mathbf{k}}\rangle &= \langle u_{n,\mathbf{k}}|\left(\frac{\mathbf{r}\times\mathbf{v}-\mathbf{v}\times\mathbf{r}}{4}\right)|u_{p,\mathbf{k}}\rangle \\ &= -\frac{i\hbar}{4}\sum_{q\neq n,p}(\sigma_3)_{qq}\left(\frac{1}{\bar{\epsilon}_{q,\mathbf{k}}-\bar{\epsilon}_{n,\mathbf{k}}}+\frac{1}{\bar{\epsilon}_{q,\mathbf{k}}-\bar{\epsilon}_{p,\mathbf{k}}}\right) \\ &\qquad \times\left[\langle u_{n,\mathbf{k}}|v_x|u_{q,\mathbf{k}}\rangle\langle u_{q,\mathbf{k}}|v_y|u_{p,\mathbf{k}}\rangle - \langle u_{n,\mathbf{k}}|v_y|u_{q,\mathbf{k}}\rangle\langle u_{q,\mathbf{k}}|v_x|u_{p,\mathbf{k}}\rangle\right].\end{aligned} \tag{S13}$$

Induced polarization

Here, we develop a phenomenological model for the electric polarization induced by the magnon orbital Nernst effect, which is intended to provide the order-of-magnitude estimation, not quantitative predictions. To obtain the magnon orbital moment profile, we adopt the drift-diffusion formalism on a two-dimensional sample. For a temperature gradient $\partial_x T$, the

spin-polarized magnon orbital moment profile is obtained by

$$\partial_t \rho_s^L + \nabla \cdot \mathbf{J}_s^L(x,y) = -\frac{\rho_s^L}{\tau}, \tag{S14}$$

where τ is the magnon orbital relaxation time, $\rho_s^L = \rho_\alpha^L - \rho_\beta^L$ is the difference of magnon orbital moment density between two magnonic modes, and

$$\mathbf{J}_s^L = -\alpha_{z,s}^L \partial_x T\,\hat{\mathbf{y}} - D\nabla \rho_s^L \tag{S15}$$

is spin-polarized magnon orbital current density with a corresponding orbital Nernst conductivity $\alpha_{z,s}^L = \alpha_{z,\alpha}^L - \alpha_{z,\beta}^L$, and a diffusion coefficient D. By assuming the uniform temperature gradient and solving the bulk equation (S14) with the boundary conditions $J_y^L(y=0)=0$ and $J_y^L(y=W)=0$, where W is the width of the sample, we obtain the steady-state solution

$$\rho_s^L(x,y) = \rho_s^L(y) = \alpha_{z,s}^L \frac{\tau \partial_x T}{\lambda \sinh\left(\frac{W}{\lambda}\right)}\left[\cosh\left(\frac{W-y}{\lambda}\right) - \cosh\left(\frac{y}{\lambda}\right)\right] = \alpha_{z,s}^L \frac{\tau \sinh\left(\frac{W-2y}{2\lambda}\right)}{\lambda \cosh\left(\frac{W}{2\lambda}\right)}\partial_x T, \tag{S16}$$

where $\lambda = \sqrt{\tau D}$ is the magnon orbital diffusion length. Fig. S1(a) shows the exponential decay of the magnon orbital moment accumulation.

Using this, we compute the electric potential due to the magnon orbital accumulation in the following method. For the magnonic spin current, we can invoke

$$\mathbf{P} = -\frac{ea}{E_{\mathrm{SO}}}\mathbf{e}_{12} \times \mathbf{I}_s, \tag{S17}$$

to compute the induced polarization since the characteristic time scale of the magnon is generally much longer than that of the electron hopping process. The spin current carried

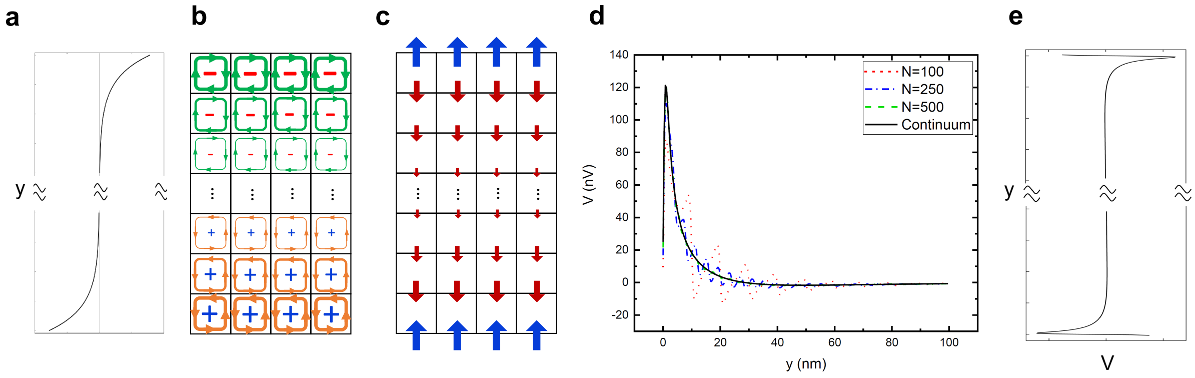

Figure S1: **a** Magnon orbital accumulation profile. **b** Schematic illustration of magnon current circulation (green and orange) and the polarization charge (red and blue) due to the magnon orbital accumulation. **c** Schematic illustration of the electric polarization. **d** Electric potential profile in the vicinity of $y = 0$ edge. Here, N denotes the number of cells along the y-direction, with a total length of 1 μm. **e** Schematic representation of the electric potential profile.

by a single magnon is given by $\mathbf{I}_s = -S(v/a)\hat{\mathbf{z}}$, which leads to the electric polarization

$$\mathbf{P} = \frac{eS}{E_{\mathrm{SO}}}(\mathbf{v} \times \hat{\mathbf{z}}), \tag{S18}$$

where $S = \pm\hbar$ is the magnon spin and $\mathbf{v} = v\,\mathbf{e}_{12}$ is the magnon velocity. Then, a magnon with $S = \hbar$ moving in a velocity $\mathbf{v}_l \equiv \mathbf{v}(\mathbf{r}_l)$ at $\mathbf{r}_l$ induces an electric dipole

$$\mathbf{P}_l \equiv \mathbf{P}(\mathbf{r}_l) = \frac{e\hbar}{E_{\mathrm{SO}}}(\mathbf{v}_l \times \hat{\mathbf{z}}), \tag{S19}$$

and the corresponding electric potential

$$V(\mathbf{r} - \mathbf{r}_l) = \frac{\mathbf{P}_l \cdot (\mathbf{r} - \mathbf{r}_l)}{4\pi\epsilon_0 (r - r_l)^3}. \tag{S20}$$

For a simple estimation, we consider that the sample consists of square unit cells with lattice constant a and assume that the magnons circulate along the lattice bonds of the each cell with a fixed speed [see Fig. S1(b)], for example, magnon revolves on k-th unit cell with a fixed speed v_k. Fig. S1(c) shows schematic illustration of the resulting electric polarizations

by Eq. (S19). We first calculate the electric potential of single square cell in the presence of the spin-polarized magnon orbital moment. If we consider a magnon as a point-like particle, the magnon's orbital moment is $va/2\,\hat{\mathbf{z}}$, where $a/2$ is the radius of orbital motion and v is the velocity. To caputure the position dependence of the magnon orbital distribution [Eq. (S16)] in the lattice model, we take cell averaged value of $\rho_s^L(\mathbf{r})$ and write it as $\bar{\rho}_s^L(\mathbf{r}'_k)$ where r'_k is position center of k-th cell. By replacing the magnon orbital moment $va/2$ to $\bar{\rho}_s^L(\mathbf{r}'_k)a^2$, we obtain the electric potential due to the magnon orbital moment on k-th cell,

$$\begin{aligned} V_k^{\text{cell}}(\mathbf{r}-\mathbf{r}'_k) &= \int_{C_k} \frac{dr''}{4a} \frac{e\hbar(\mathbf{v}_k \times \hat{\mathbf{z}})\cdot(\mathbf{r}-\mathbf{r}'')}{4\pi\epsilon_0 E_{\text{SO}}|\mathbf{r}-\mathbf{r}''|^3} \\ &= \int_{C_k} \frac{dr''}{4a} \frac{e\hbar\bar{\rho}_s^L(\mathbf{r}'_k)a^2(\hat{\mathbf{v}} \times \hat{\mathbf{z}})\cdot(\mathbf{r}-\mathbf{r}'')}{2a\pi\epsilon_0 E_{\text{SO}}|\mathbf{r}-\mathbf{r}''|^3} \\ &= \frac{e\hbar\bar{\rho}_s^L(\mathbf{r}'_k)}{8\pi\epsilon_0 E_{\text{SO}}} f(\mathbf{r}-\mathbf{r}'_k), \end{aligned} \tag{S21}$$

where C_k is the closed path along lattice bonds of the unit cell centered at $\mathbf{r}'_k$. Here we take the line average of the lattice bonds, because we assume the magnons circulate along the lattice bonds of the cell with fixed speed. The function f is resulted from the integration along the unit cell, *i.e.*,

$$\begin{aligned} f(x,y,z) = \sum_{p,q=\pm1} (-1)^{(p+q)/2} &\left(\frac{1}{(x+pa/2)^2+z^2} + \frac{1}{(y+qa/2)^2+z^2} \right) \\ &\times \frac{(x+pa/2)(y+qa/2)}{\sqrt{(x+pa/2)^2+(y+qa/2)^2+z^2}}. \end{aligned} \tag{S22}$$

Eq. (S22) is in a form of the summation of the function $f_e(x,y,z) = \left(\frac{1}{x^2+z^2} + \frac{1}{y^2+z^2}\right)\frac{xy}{\sqrt{x^2+y^2+z^2}}$ shifted onto the each lattice site of the cell with the alternating sign. By summing up the local contributions from each cell [as given in Eq. (S21)] across the entire sample, we obtain the electric potential profile

$$V(\mathbf{r}) = \sum_k V_k^{\text{cell}}(\mathbf{r}-\mathbf{r}'_k) \tag{S23}$$

depending on number of cells along the y-direction [see Fig. S1(d) and (e)].

Fig. S2 shows the potential profiles in the continuum limit for various values of magnon orbital relaxation time τ and magnon orbital diffusion length λ.

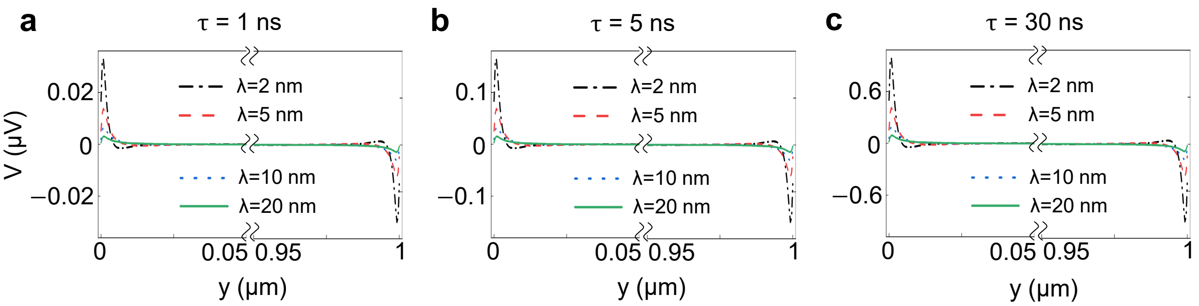

Figure S2: Induced electric potential profiles for **a** $\tau = 1$ ns, **b** $\tau = 5$ ns, and **c** $\tau = 30$ ns.

Longitudinal spin current

Here we compute the longitudinal spin current with a sample length L in the case where the magnon spin current is blocked at the sample boundaries. In the presence of the temperature gradient the longitudinal spin current is given by

$$j_x^s = -\alpha_x \partial_x T - D_s \nabla \rho_s \tag{S24}$$

where ρ_s is the magnon accumulation and D_s is the magnon diffusion constant. To solve the diffusion equation, we take an ansatz $\rho_s = Ae^{-x/l_m} + Be^{x/l_m}$, where l_m is the magnon diffusion length. By solving the diffusion equation with the open boundary condition $j_x^s(0) = j_x^s(L) = 0$, we obtain

$$j_x^s(x) = -\alpha_x \partial_x T \left(1 - \cosh\frac{L-2x}{2l_m}\mathrm{sech}\frac{L}{2l_m}\right). \tag{S25}$$

In Fig. S3 we show the longitudinal spin current profiles with different values of l_m/L. When l_m/L is large, the longitudinal spin current becomes negligible, as it is effectively counteracted by the backflow spin current induced by the gradient in magnon accumulation.

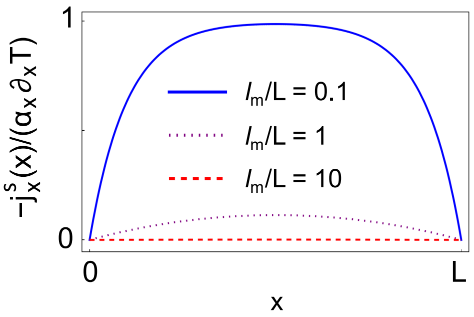

Figure S3: Longitudinal spin current profiles with different values of l_m/L.

References

(1) Zyuzin, V. A.; Kovalev, A. A. Magnon Spin Nernst Effect in Antiferromagnets. *Phys. Rev. Lett.* **2016**, *117*, 217203.

(2) Li, B.; Sandhoefner, S.; Kovalev, A. A. Intrinsic spin Nernst effect of magnons in a noncollinear antiferromagnet. *Phys. Rev. Res.* **2020**, *2*, 013079.

(3) Cheng, R.; Okamoto, S.; Xiao, D. Spin Nernst Effect of Magnons in Collinear Antiferromagnets. *Phys. Rev. Lett.* **2016**, *117*, 217202.

(4) Zhang, X.; Zhang, Y.; Okamoto, S.; Xiao, D. Thermal Hall Effect Induced by Magnon-Phonon Interactions. *Phys. Rev. Lett.* **2019**, *123*, 167202.

(5) Bhowal, S.; Vignale, G. Orbital Hall effect as an alternative to valley Hall effect in gapped graphene. *Phys. Rev. B* **2021**, *103*, 195309.

(6) Pezo, A.; García Ovalle, D.; Manchon, A. Orbital Hall effect in crystals: Interatomic versus intra-atomic contributions. *Phys. Rev. B* **2022**, *106*, 104414.

(7) Busch, O.; Mertig, I.; Göbel, B. Orbital Hall Effect and Orbital Edge States Caused by s Electrons. *Phys. Rev. Res.* **2023**, *5*, 043052.
